# Evaluating Efficiency of a Provincial Telerehabilitation Service in Improving Access to Care During the COVID-19 Pandemic

**DOI:** 10.5195/ijt.2023.6523

**Published:** 2023-05-11

**Authors:** Katelyn Brehon, Jay Carriere, Katie Churchill, Adalberto Loyola-Sanchez, Elizabeth Papathanassoglou, Rob MacIsaac, Mahdi Tavakoli, Chester Ho, Kiran Pohar Manhas

**Affiliations:** 1 Department of Physical Therapy, University of Alberta, Edmonton, Alberta, Canada; 2 Department of Electrical and Software Engineering, University of Calgary, Calgary, Alberta, Canada; 3 Allied Health Professional Practice and Education, Alberta Health Services, Alberta, Canada; 4 Department of Occupational Therapy, University of Alberta, Edmonton, Alberta, Canada; 5 Department of Medicine, University of Alberta, Edmonton, Alberta, Canada; 6 Neurosciences, Rehabilitation, and Vision Strategic Clinical Network^™^, Alberta Health Services, Alberta, Canada; 7 Faculty of Nursing, University of Alberta, Edmonton, Alberta, Canada; 8 Spinal Cord Injury Alberta, Edmonton, Alberta, Canada; 9 Department of Electrical and Computer Engineering, University of Alberta, Edmonton, Alberta, Canada; 10 Department of Community Health Sciences, University of Calgary, Calgary, Alberta, Canada

**Keywords:** Artificial intelligence, Call utilization, Machine learning, Qualitative description

## Abstract

**Scope:**

Early in the COVID-19 pandemic, community rehabilitation stakeholders from a provincial health system designed a novel telerehabilitation service. The service provided wayfinding and self-management advice to individuals with musculoskeletal concerns, neurological conditions, or post-COVID-19 recovery needs. This study evaluated the efficiency of the service in improving access to care.

**Methodology:**

We used multiple methods including secondary data analyses of call metrics, narrative analyses of clinical notes using artificial intelligence (AI) and machine learning (ML), and qualitative interviews.

**Conclusions:**

Interviews revealed that the telerehabilitation service had the potential to positively impact access to rehabilitation during the COVID-19 pandemic, for individuals living rurally, and for individuals on wait lists. Call metric analyses revealed that efficiency may be enhanced if call handling time was reduced. AI/ML analyses found that pain was the most frequently-mentioned keyword in clinical notes, suggesting an area for additional telerehabilitation resources to ensure efficiency.

The COVID-19 pandemic triggered the rapid adoption of telehealth services to advance care continuity while minimizing risks of COVID-19 infection (Brehon et al., 2022; Wosik et al., 2020). Telehealth services offer benefits to patients and the health system. Patients benefit from increased accessibility and comfort as they can receive care in their homes, decreased wait times, and lower financial and time costs of travelling to and from appointments (Gajarawala & Pelkowski, 2021; Lifeline Research Foundation, 2013). The health system benefits from decreased costs of providing in-person services, fewer unnecessary emergency department visits, and increased efficiency of service provision (Gajarawala & Pelkowski, 2021).

Research demonstrates, at minimum, equivalence between telerehabilitation, the virtual delivery of rehabilitative services, and usual, in-person care in various contexts, including care for musculoskeletal conditions, inflammatory arthritis, and orthopedic surgery (Alberta Health Services Provincial Rehabilitation Forum, 2016; Lee et al., 2018; Pastora-Bernal et al., 2017; Seron et al., 2021; Taylor-Gjevre et al., 2018). Telerehabilitation has been perceived by patients as a convenient way to receive services (Buabbas et al., 2022) with patient satisfaction found to be high (Amin et al., 2022; Johansson & Wild, 2011; Moffet et al., 2017; Tousignant et al., 2011; Tsvyakh & Hospodarskyy, 2017). Physiotherapists also reported satisfaction with telerehabilitation (Amin et al., 2022; Tousignant et al., 2011) and note perceived improvements in patient access to services and reduced wait times (Buabbas et al., 2022).

In April 2020, a provincial health system in Canada sought to mobilize telerehabilitation to respond to service inconsistencies and variable social distancing mandates during the COVID-19 pandemic. As the first of its kind in Canada, this telerehabilitation service, known as the Rehabilitation Advice Line (RAL), provides wayfinding and self-management advice to people experiencing disabilities. It initially set its scope to address functional concerns related to musculoskeletal conditions, neurological conditions, and/or post-COVID-19 recovery needs. The service is run by physiotherapists and occupational therapists and is available during normal business hours.

We reported on the evaluation of the telerehabilitation service's impact (Brehon et al., 2022). Usability measurements showed that callers were satisfied, corroborating literature from pre-pandemic contexts (Brehon et al., 2022). However, the satisfaction and acceptability of the service did not supplant preferences for in-person visits (Brehon et al., 2022). In addition, the population included in this study reported lower quality of life compared with the provincial population, conflicting with pre-pandemic research, which may be due to added stressors associated with the pandemic (Brehon et al., 2022).

Given the modicum of impact identified, it is important to assess the service's efficiency in the context of evolving rehabilitation needs in our post-pandemic realities. We are defining efficiency based on the Health Quality Council of Alberta's Quality Matrix for Health: “resources are optimally used in achieving desired outcomes” (Health Quality Council of Alberta, 2005). Efficiency typically connotes cost savings. However, we did not complete cost savings analyses as we were focused on understanding efficiency of the service near inception based on caller perspectives and call metrics rather than finances. It was our goal that the findings from the current study would be translated into alterations in service provision to ensure long-term sustainability and efficient use of health system resources. Consequently, the current study's aims were:

Aim 1: To understand caller perspectives on areas where the service is currently operating efficiently as well as perceived areas for improvement thus providing insights on sustainability.

Aim 2: To understand the implications of service utilization and call patterns on efficiency of the service, which subsequently effects sustainability.

## Methods

We used a multiple methods design. We briefly describe our methods (detailed methods published elsewhere (Brehon et al., 2021)). We used qualitative interviews to address Aim 1 supplemented with secondary data analyses of call metrics and narrative analyses of clinical notes using Artificial Intelligence/Machine Learning (AI/ML) to address Aim 2.

The University of Alberta Health Research Ethics Board approved this study (Pro00102178). A waiver of consent was obtained for secondary data analysis. All other participants provided informed written consent.

### Study Population

The study population included adult callers who accessed the telerehabilitation service within the first six months of operation. Callers included patients or caregivers (wherein the patient could not provide consent, or the caregiver was the legal guardian). Inclusion criteria for qualitative interviews were: age 18+ years; able to communicate in English; willing to participate in the research; and able to provide informed consent. There were no inclusion or exclusion criteria for secondary analyses.

### Aim 1: Caller Perspectives on Service Efficiency

#### Methodological Framework

We used in-depth interviews to clarify caller perceptions of service efficiency. We used Sandelowski's framework for qualitative description as a methodological framework to ground our qualitative work (Sandelowski, 2000). Qualitative description studies produce findings that are “data near” (Sandelowski, 2010) meaning that the findings are closer to the data than they would be in an interpretive description study, for example. This methodology is helpful when trying to understand the current state of a phenomenon as it focuses on the who, what, and where of events and experiences and includes moderately-structured, open-ended questions (Sandelowski, 2000). Rather than reading *into* the lines, data analysis for qualitative description involves “reading *of* the lines” (Sandelowski, 2010). However, as Sandelowski (2010) notes, there is still an interpretive element to qualitative description as the individual conducting the analysis cannot completely remove themselves and their epistemologies. In other words, there is an element of interpretation involved in every qualitative analysis; what differs is the level of interpretation undertaken. For the purposes of the current study, minimal interpretation of caller quotations was utilized as the goal was to describe current factors contributing to efficiency and areas of improvement, rather than reading *into* what the callers were saying to uncover underlying facets (Sandelowski, 2010).

#### Recruitment

We aimed to recruit 8-12 callers. Callers who consented to future contact at the end of their call interaction were sent a follow-up survey three months following the call. This survey assessed the impact of the telerehabilitation service and findings have been reported elsewhere (Brehon et al., 2022). The survey was sent at three-months post-interaction as we were interested in understanding some of the longer-term impacts of the service. At the end of the survey, callers were asked if they were interested in participating in an interview about their experience. We utilized this convenient method of recruitment for feasibility purposes (i.e., allowed us to recruit from one pool of potential participants versus asking clinicians at the service to recruit at two time periods) as well as because we were interested in understanding longer-term perceptions of efficiency, thus contributing to sustainability. We contacted callers to organize interviews by phone within one week following completion of a three-month follow-up survey (Brehon et al., 2022).

#### Data Collection

Callers participated in one-time, semi-structured interviews. Interview questions addressed how the caller first heard about the service, call experiences, what had happened since the call interaction regarding the issue they called about, general perceptions of the service, and thoughts on what would be important to sustainability of the service. The full interview guide can be found in Appendix A.

Due to limitations posed by the COVID-19 pandemic, the virtual nature of the research team, and the provincial nature of the service, interviews took place over the phone or videoconferencing software. Throughout the interview, the interviewer probed for further details as necessary. Interviews were audio-recorded and confidentially transcribed verbatim.

#### Data Analysis

Our analysis was informed by Braun and Clarke's six phases of reflexive thematic analysis (Braun & Clarke, 2006, 2021). To ensure comprehensiveness and peer audit, each transcript was coded independently by two members of the research team. The analysis process began by both research team members becoming familiar with the data by reading all transcripts and reviewing them for accuracy. Initial codes were generated inductively. The research team then met to collate codes and ideas into initial themes. The codes and initial themes were then reviewed and considered in relation to one another and collapsed or expanded based on patterns of meaning. Themes and sub-themes were then defined to ensure each theme and sub-theme were unique and did not overlap. Analysis continued while the final report was being drafted.

We promoted qualitative rigour by using an audit trail of decisions for accountability, employing open-ended questions to prioritize participant voices, ensuring thick description for fidelity of participant voice, and implementing collaborative coding to expose biases during analysis and ensure credibility of interpretations.

### Aim 2: Improving Efficiency Through Analysis of Call Metrics and Clinical Notes

We used secondary data analyses of call metrics and AI/ML analyses of clinical notes to explore call utilization, call quality, and the population accessing the service during the evaluation period.

We used de-identified data of the entire population of callers, rather than a sampling or criterion-based approach. Data was electronically captured at each call by the service. The variables analyzed to address this aim are outlined in Table 1. Data were analyzed descriptively using IBM SPSS 26 (Chicago, IL). We calculated the total, mean, and standard deviations (SD) for all call utilization variables and caller variables 1-3 (Table 1).

**Table 1:** Call Metrics and Caller Data Utilized for Secondary Analyses and AI/ML Analyses, Respectively

	Variables	Details
**Call Metrics**	1. Number of calls in total and per week	Number of incoming calls
	2. Number of call backs in total and per week	Number of outgoing calls
	3. Number of abandoned calls in total and per week	Calls that were abandoned by providers due to the call being disconnected
	4. Average talk time in total and per week	Talking time is the amount of time clinicians spent with a caller on the phone.
	5. Average handling time in total and per week	Handling time is the time it took for the clinician to complete the clinical note and send any information to the caller.
	6. Total average call length in total and per week	Measures in minutes
	7. Average call hold time in total and per week	Time the caller spent on hold prior to connecting with a provider and/or once connected with a provider
**Caller Variables**	1. Caller age	Caller's age if a person was phoning in for themselves, or the age of the person that the call was about if another person phoned in on their behalf
	2. Caller gender	Male, female, or undisclosed
	3. Caller healthcare zone	The actual zone in which the caller lives. This was to break down the analysis into the three healthcare zones (Calgary zone, Edmonton zone, and the combined Rural zones)
	4. Reason for phoning the RAL, rehabilitation assessments, patient concerns, and the information/services provided	Contained in free-text clinical notes

AI/ML technology was used to provide insight into a caller's reason for phoning the service (caller variable 4 in Table 1). AI/ML tools, such as natural language processing (NLP), have previously been underutilized but are promising for the evaluation of telerehabilitation initiatives (Carriere et al., 2021; Tavakoli et al., 2020). AI/ML technologies consist of a broad range of tools that provide insight and modelling of complex phenomena. AI/ML analyses were processed using the Apache cTakes NLP system (Savova et al., 2010). Data underwent NLP preprocessing to extract keyword information from the free-text clinical notes regarding call history, action, and disposition as outlined in Table 2.

**Table 2:** Clinical Text Information Input into the NLP System for Analysis

Content within Clinical Notes	
**History**	The caller's relevant medical history, including existing chronic or acute musculoskeletal, neurological, or other conditions.
**Action**	The formal and informal assessment provided by the RAL clinician, including activities of daily living, standard rehabilitation assessment metrics, social conditions, and mental health concerns.
**Disposition**	The care plan and services provided by the RAL clinician, including service referrals, scheduling follow-up phone calls/emails, referral to online information (e.g. AHS website).

After c Takes NLP preprocessing, the input unstructured clinical notes were in a parsed machine-readable format that includes part-of-speech tagging, healthcare keyword classification, and mapping to Unified Medical Language System (UMLS) identifiers (Bodenreider, 2004). Further AI/ML analysis was performed on the text, using context clues from the part-of-speech tagging and UMLS identifiers, to determine the most salient, and common, keywords mentioned during caller interactions.

These keywords were then reduced into a simplified keyword list for grouping the type of call into three different categories: musculoskeletal concerns, neurological conditions, or post-COVID-19 rehabilitation needs. For musculoskeletal and neurological calls, a list of keywords associated with musculoskeletal or neurological conditions was manually compiled. The notes captured during each call were processed and the number of musculoskeletal terms compared to neurological terms mentioned in the call were counted. The call was classified as musculoskeletal, for example, if a larger proportion of the keywords within the call were musculoskeletal-related. In the case that an equal number of musculoskeletal and neurological keywords were mentioned, the call was classified as undefined. For post-COVID-19 calls, clinical notes were manually searched to determine whether they related to post-COVID-19 rehabilitation needs. The full AI/ML processing pipeline can be found in Appendix B.

## Results

### Aim 1: Caller Perspectives on Service Efficiency

Ten callers discussed their thoughts on the service's efficiency by interview. All interviews were conducted at least three months following the original call interaction. The interviews were 9 to 27 minutes long. All interviews were conducted by the same experienced interviewer, who is trained in qualitative methods.

Two key themes related to efficiency emerged from the interviews: (1) professional communication, and (2) opportunities to improve service efficiency. Theme one broached communication during, and after, the call and related perceptions of the service's efficiency. In theme two, opportunities to improve service efficiency included bridging the care gap (i.e., providing a service for individuals on waitlists), improving access to care for individuals living in rural communities and during COVID-19, and the importance of advertising the service.

#### Theme 1: Professional Communication

Callers spoke to the professional communication that they experienced during, and after, the call, which cultivated trust and perceptions of the legitimacy of the telerehabilitation service and subsequently improved access to care. These feelings equated to the service being perceived as an efficient way to receive self-management and wayfinding advice.

**Professionalism and Communication During the Call**. Callers felt that the clinicians providing the service were caring, knowledgeable, thorough, and professional. These qualities were demonstrated through a balance of clinicians' compassion for callers' situations as well as their ability to efficiently assess callers' issues without physically seeing them. Callers felt that clinicians were cognizant of their comfort during the phone assessment. Callers left the call feeling cared for and with an ability to return if needed. Callers did not feel rushed during conversations. Most callers appreciated that the service existed and that it gave them an avenue to access rehabilitation providers over the phone.

*“… the specific woman I talked to was really knowledgeable I thought … she listened to what I was saying so I like that … I like the idea of … talking to someone who is … a professional you know?” (Caller 8)*

The challenge of setting realistic expectations was discussed by callers. For example, one caller who contacted the service for a caregiving-related issue mentioned how it did not give *“real, tangible, actual help”* (Caller 10) for a complex challenge. Another caller discussed how the service did not get to the root cause of their issue, but they acknowledged that this might not have been achieved in-person either. While the telerehabilitation service and its clinicians were generally well-received, moving forward, advertisements and call introductions should enable callers to align their expectations with service capabilities and potential limitations thus ensuring efficiency of use.

*“… it was just a really uplifting feeling to find out there's someone who could help me but then even after I got the information it was just a real let down because … it didn't provide me with real, tangible, actual help… they were really good suggestions but … nothing came out of it.” (Caller 10)*

**Communication After the Call.** Callers were generally pleased with the communication and follow-up that they received after the call, and this helped to further develop a therapeutic relationship. Communication after the call included scheduled follow-up, the ability to call back whenever they needed, and/or emailed resources. Callers felt that the emailed resources were easy to follow and access while promoting perceptions of legitimacy and professionalism of the telerehabilitation service. Callers discussed how the materials aligned with the phone conversation. This link between the call and the follow-up helped to efficiently support care continuity by ensuring that the caller had all the information that they needed to self-manage.

*“I just came away feeling like she told me what I needed to know and … basically if I needed any more help or anything else I would just get back to her … the email handouts they sent me were excellent … I was surprised how good they were as a matter of fact.” (Caller 6)*

#### Theme 2: Opportunities to Improve Service Efficiency

Callers spoke about potential ways to improve the service, therefore increasing its efficiency. They suggested that the service could be used to provide interim rehabilitative care while individuals were on waitlists (i.e., for hip or knee replacement) or when there were long gaps in their care journey. They advocated for the service to be used to increase access to care during the COVID-19 pandemic and for individuals living in rural areas. For example, one caller noted how the service could be helpful for rural populations as it would be an efficient way to overcome geographical and mobility challenges. Another caller also suggested the development of a website providing a diagram of the body to allow callers to use the same language as clinicians during phone assessments, therefore improving efficiency of assessment and when providing clinical recommendations.

*“I had a knee injury … and finally decided to seek medical help but COVID was going on … [so] I phoned the physio department, they only have one where I live, they said we're not taking anybody but we'll put you on the list and you're number 45 on the list [and] I knew I wasn't going to see anybody any time soon so then I asked the question, is there any online help [and] they offered up the phone number for [the telerehabilitation service]” (Caller 2)*

Callers recognized that while the telerehabilitation service was helpful, lack of knowledge about its availability would lead to lack of benefit. In order to ensure optimal use of the service and therefore justify its existence, callers recognized the need for concerted advertising efforts to promote public awareness of the service's availability and scope. Most callers supported diverse advertising strategies as a variety of advertising strategies would promote inclusivity of various demographics. Callers suggested flyers in their local grocery store or hockey rinks; news stories on news apps or television; social media marketing; search engine optimization; and advertising on government-run COVID-19 webpages.

*“I would advertise that line … if they've got any … money for it because … it's a good line and to just let people know it's available, I think it could help a lot of people.” (Caller 4)*

### Aim 2: Improving Efficiency Through Analysis of Call Metrics and Clinical Notes

#### Call Metric Analysis

There were 537 clinical call interactions between May 12, 2020 and October 31, 2020. Callers identified as male (n=201, 37.4%), female (n=321, 59.8%), and did not identify their gender (n=15, 2.8%). The mean (standard deviation, SD) age of callers was 55.33 (18.13) years.

**Figure 1:**
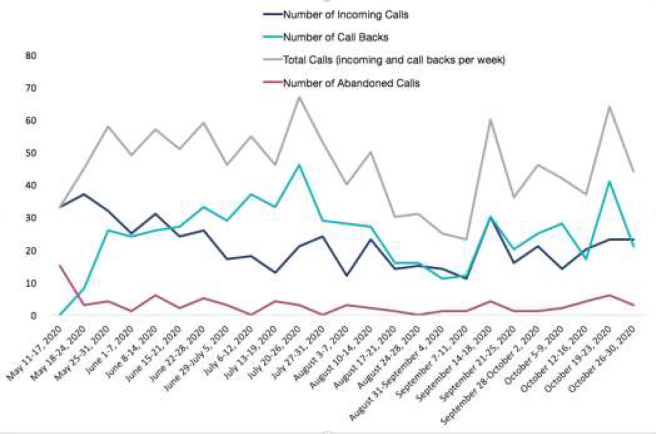
Number of Calls, Call Backs, Total Calls, and Abandoned Calls by Week

Call metric data separated by week can be found in Appendix C. Table 3 outlines the total, mean, and SD values for number of calls, call backs, and abandoned calls, as well as mean times for talking, handling, on-hold, and in-total. Number of calls, call backs, and abandoned calls by week can be found in Figure 1. The mean (SD) talk, handling, call, and hold time were 14.75 (3.87) minutes, 22.23 (8.37) minutes, 36.98 (10.60) minutes, and 3.11 (1.55) minutes, respectively.

**Table 3:** Call Metric Data between May 12, 2020 and October 31, 2020

	Number of Calls	Number of Call Backs	Number of Abandoned Calls	Average Talk Time (minutes)	Average Handling Time (minutes)	Average Call Time (minutes)	Average Hold Time (minutes)
**Minimum**	11	0	0	5.20	14.73	23.12	0.33
**Maximum**	37	46	15	20.35	46.35	62.73	6.37
**Median**	21	26	3	15.62	20.43	35.83	3.03
**Total**	537	610	75	368.74	555.73	924.47	77.67
**Mean (per week)**	21.48	24.4	3	14.75	22.23	36.98	3.11
**Standard Deviation (per week)**	7.21	10.39	3.06	3.87	8.37	10.60	1.55

#### Clinical Note Analysis

AI/ML was used to analyze 412 eligible calls. AI/ML analyses were limited to interactions where the clinician opened a clinical note. Excluded call interactions were those that were brief (< 5 minutes) as these call interactions were deemed non-clinical in nature (i.e., leaving a voicemail, not documented in a memo). The dataset from AI/ML analyses was similar in terms of age and geographical distribution of the total call volume during this period. For this subset of callers, the mean (SD) caller age across the province was 54.5 (17.4) years old, 251 callers were female (60.9%), 161 callers were male (39.1%), and the mean (SD) call duration was 53.1 (27.2) minutes (graphical details in Appendix D).

Calls analyzed with AI/ML were distributed geographically with 330 (80.1%) calls from urban zones and 82 (19.9%) calls from rural zones. The distribution of zone population, number of calls, call duration, caller age, and caller gender can be found in Table 4.

**Table 4:** Number of Calls, Caller Age, and Call Duration by Zone for Calls Requiring a Clinical Note between May 12, 2020 and October 31, 2020

Healthcare Region	Urban Zones	Rural Zones
**Total Zone Population, in millions of people (percentage of total)**	2.70 (71.0%)	1.28 (29.0%)
**Number of Calls (percentage of total)**	330 (80.1%)	82 (19.9%)
**Call Duration (standard deviation)**	51.1 (29.0) minutes	55.0 (25.3) minutes
**Caller Age (standard deviation)**	55.5 (17.7) years	53.5 (17.0) years
**Caller Gender (percentage with respect to region)**	204 female (61.8%) 126 male (38.1%)	47 female (57.3%) 35 male (42.7%)

There were 371 musculoskeletal calls (90%), 21 neurological calls (5.1%), 3 post-COVID-19 calls (0.7%), and 17 undefined calls (4.1%) as shown in Appendix D. Table 5 shows the top 10 keywords as well as the types of symptoms and disorders mentioned, giving the primary reasons for calling. Pain was the most significant keyword, mentioned by 76.5% of callers to the telerehabilitation service.

**Table 5:** Top Keywords and Reason for Call Analysis during Period (May 12, 2020 – October 31, 2020)

Key Word	Number of Calls in which Keyword was Mentioned (Percentage of AI/ML Calls)
Pain	315 (76.5%)
Injury	99 (24.0%)
Falls	84 (20.3%)
Sleep	69 (16.7%)
Swelling	69 (16.7%)
Numbness	67 (53%)
Fracture	53 (48%)
Sore to Touch	48 (11.8%)
Ability to Balance	47 (11.5%)
Arthritis	37 (9.1%)

## Discussion

We sought to clarify the efficiency of a novel telerehabilitation service at improving access to care using multiple methods: semi-structured interviews with callers, secondary analyses of call metrics, and AI/ML analyses of clinical notes.

Positive provider qualities strengthened therapeutic relationships. This finding builds on our previous quantitative work evaluating the impact of the telerehabilitation service (Brehon et al., 2022). In this study, we found that the RAL was perceived by callers as highly useable and overall, the majority of callers (94.4%) were satisfied with the service (Brehon et al., 2022).

Qualitative results from the current study revealed that satisfaction with the service resulted mainly from efficient communication and relationship-building during and after the call which improved access to care. In a systematic review (n=45 articles) studying interpersonal provider attributes in provider-patient interactions during telehealth care delivery, rapport building was found to be an essential aspect of telehealth interactions (Henry et al., 2017). Rapport was built when providers were caring, listened, communicated efficiently, were competent, and collaborated with the patient (Henry et al., 2017) which is similar to what was discussed by callers in the current study.

While callers seemed generally satisfied with the service, they also highlighted some suggestions to improve efficiency. Their suggested improvements included: (1) multi-pronged, age-specific marketing strategies to promote the service; (2) the service should provide referrals to the clinics they recommend; (3) there should be a website available to callers so that when they are on the call, they can refer to the body diagram on the website to ensure they are using a common language (i.e., calling a body part the same name as the clinician) when describing their challenges to clinicians; (4) the service should be utilized to help manage waitlists; and (5) the service should provide call backs, which is an idea that has since enhanced delivery. A study analyzing the impact of telehealth communication provided by nurses suggested that targeted marketing efforts were critical for successful communication via telehealth (Barbosa & Silva, 2017). Similarly, in the systematic review discussed previously (n=45 articles), the authors noted that to obtain quality patient and provider relationships, there needs to be high levels of access to telerehabilitation initiatives, which could be improved via clear communication about the initiative (Henry et al., 2017). These findings suggest that concerted marketing efforts are critical to the success of telehealth initiatives. In a pre-post study exploring the impact of telehealth strategies on waitlists, Gadenz et al. (2021) found that referral management using telehealth decreased wait times by promoting more coordinated care (Gadenz et al., 2021). This may suggest that if the telerehabilitation service evaluated in the current study helped to manage referrals to certain clinics, such clinics may have reduced wait times and therefore improved efficiency of service provision and access to care. Similarly, a retrospective cohort study found that telemedicine for primary care reduced waitlists for specialty consultations with sicker patients receiving care quicker (Pfeil et al., 2020). Further, a scoping review (n=27) found that telehealth interventions, such as electronic consultations and image-based triage, can reduce wait times (Caffery et al., 2016). While these studies did not analyze a phone-based service nor were conducted in the context of rehabilitation, they suggest that if the telerehabilitation service from the current study was employed as a method to provide referrals and subsequently reduce wait times, it may have positive effects on the efficiencies of other services.

Call metric analyses showed that most callers were female and in their mid-50s. Mean call handling time was longer than the actual talk time that the clinician spent with a caller on the phone. This finding provides an actionable learning: handling time must be reduced to ensure efficiency. A study analyzing the operational determinants of caller satisfaction for call centers found a significant negative correlation between average work time following calls and caller satisfaction: as average work time following calls was decreased, caller satisfaction was improved (Feinberg et al., 2000). While this study is not specific to a health care setting, given that the telerehabilitation service uses a call center structure, reduction in handling time may improve caller experience by ensuring clinicians are available to take more incoming calls subsequently reducing wait times and therefore contributing to the overall efficiency of the service. Higher mean handling time in the current study likely resulted directly from the early timing of the evaluation (shortly following inception) as clinicians were still learning the documentation processes required following a call. A follow-up evaluation would inform whether the learning curve was overcome to reduce handling times.

The AI/ML sub-analyses resulted in similar demographic characteristics as the full study population. These analyses revealed that the majority of calls were identified as musculoskeletal-related with pain being the most frequently mentioned keyword. These findings provide insight into areas that the telerehabilitation service may want to devote additional marketing, (i.e., make the community aware that rehabilitation can assist with more than musculoskeletal issues) and resources (i.e., develop new or updated self-management resources for pain management) in order to improve this service's efficiency. However, AI/ML analyses were limited by the fact that the clinical notes were unstructured. More structured notes with identifiable features (e.g., following the subjective, objective, assessment, and plan format) and a larger dataset would allow for more rigorous AI/ML analyses to be conducted. In their review (n=22 articles) of statistical and ML methods for modelling cancer risk, Richter and Khoshgoftaar (2018) support the need for structured clinical notes as they: (1) help build high-impact AI/ML models that can be generalized to a diverse population, and (2) are more easily de-identified for data analysis purposes (Richter & Khoshgoftaar, 2018). While NLP preprocessing was used to try to overcome the barrier of not having structured clinical notes, it was limited by the time it took to conduct as well as the need to manually compile a list of relevant key words for each clinical condition. Structured clinical notes would allow for more rigorous analyses which would provide more reliable details of callers' reasons for contacting the service and allow the service's resources to be tailored appropriately, therefore contributing to efficiency.

## Limitations

Study limitations are recognized. First, our study may have been impacted by selection and recall bias. There may be commonalities between those that chose not to consent for further contact following their call and were therefore not able to be contacted for recruitment. Recall bias may have also impacted participants as we interviewed them at least three months following their call interaction. This may be why the interviews were quite short (9–27 minutes). However, this choice of interview timing was intentional as we were interested specifically in understanding their insights on efficiency of the service at this time point. It was also more feasible to recruit following the impact survey (results published elsewhere: Brehon et al., 2022) as it meant the clinicians at the service did not have to recruit a second population for us to sample from. Second, as mentioned, the AI/ML analyses were limited by sample size and the unstructured nature of the clinical notes. In general, data-driven AI/ML systems require a sufficiently large input data set to function properly. However, the current data set was limited due to the novel nature of the telerehabilitation service and the early timing of the study.

## Conclusion

In conclusion, we aimed to clarify efficiency of a novel telerehabilitation service. By utilizing multiple methods including interviews, secondary data analyses, and AI/ML analyses we were able to evaluate the service within a pandemic context. Qualitative analyses illuminated that the telerehabilitation service has the potential to positively impact rehabilitation access for rural areas, during the COVID-19 pandemic, and for those waiting to access other services (i.e., hip or knee replacements, in-person rehabilitation services). Call metric analyses outlined areas for improvement to ensure efficiency of the service: handling time after the call was longer than the time clinicians spent on the phone with callers. This finding suggests an area for improvement to ensure service efficiency. AI/ML analyses revealed areas that the service may want to devote more resources to (i.e., pain and/or musculoskeletal rehabilitation). Given the evolving literature base on Long-COVID sequelae, future research should focus on evaluating the effectiveness of the service in meeting the rehabilitative needs of the Long-COVID population within this Canadian context.

## Figures and Tables

**Figure B1:**
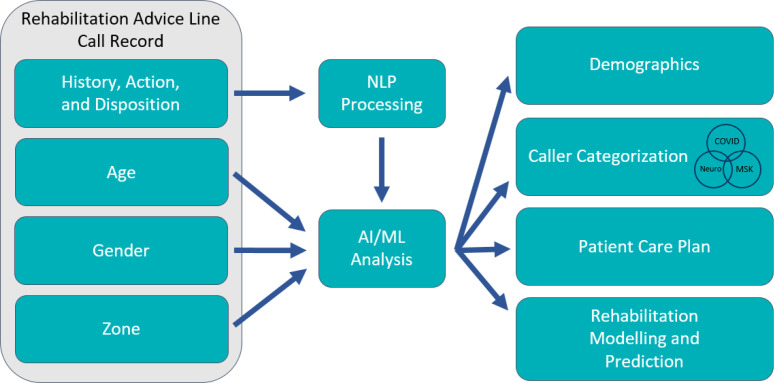
Outline of Analysis Performed on Caller Data by the Combined AI/ML System

**Figure D1:**
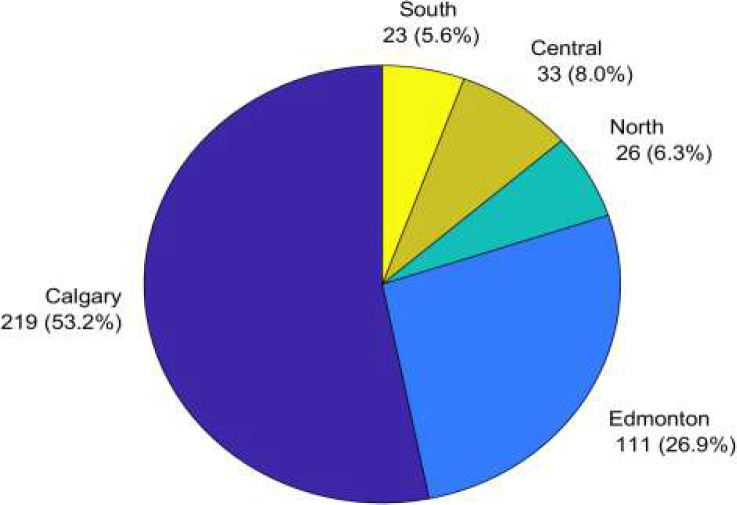
Call Volume by Healthcare Zone for AI/ML Analyzed Calls During Period (May 12, 2020 – October 31, 2020)

**Figure D2:**
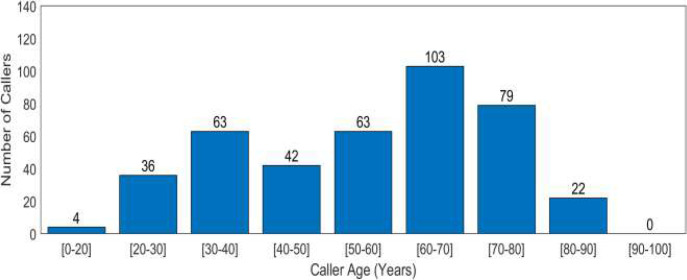
Caller Age for AI/ML Analyzed Calls During Period (May 12, 2020 – October 31, 2020)

**Figure D3:**
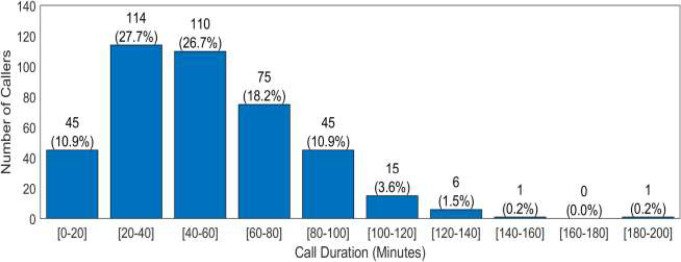
Call Duration for AI/ML Analyzed Calls During Period (May 12, 2020 – October 31, 2020)

**Figure D4:**
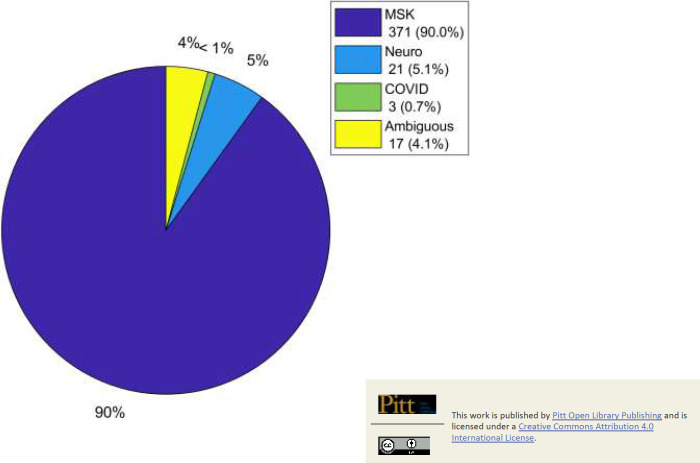
Call Type Found Using AI/ML Analysis of Calls During Period (May 12, 2020 – October 31, 2020)

## Appendix A Rehabilitation Advice Line Interview Guide

We are evaluating the early phases in the Rehabilitation Advice Line so that we can make improvements, continue successes, and determine its long-term feasibility. We greatly appreciate your time and your willingness to share your experience. Please note that you do not have to answer any question if you do not want to, and we can stop at any time.

1. How did you first hear about the Rehabilitation Advice Line?
2. Please describe your experience using the Rehabilitation Advice Line?
  Possible probes:
  - What went well?
  - What did not appear to go well?
  - What did you perceive as barriers to the support you need?
  - What did you perceive as enablers to the support you need?
  - How did you find these specific aspects of the call: **Information sharing? Clinician-patient communication**? **Questions asked? Time available? Ability to discuss your concerns? Resources available? How your concerns were dealt with?**
3. With respect to the concern that you called the Rehabilitation Advice Line about, what has happened since your first call?
4. Please describe your perception of the Rehabilitation Advice Line?
  Possible probes:
  - How do you feel about the hours?
  - How do you feel about the introductory questions?
  - What do you perceive as barriers to this service's success or feasibility?
  -What did you perceive as enablers of this service's success or feasibility?
5. What do you think will be important to the sustainability of the Rehabilitation Advice Line in the long-term?
  Possible probes:
  - What to change?
  - What to maintain?
  - Do you think the line should be maintained? Why or Why not?
6. Would you like to share anything else about your experience with the Rehabilitation Advice Line?

## Appendix C Call Metric Analysis

**Table C1:** Call Metric Data for First 5.5 Months Post-launch (May 12, 2020 – October 31, 2020)

Week	Number of Calls	Number of Call Backs	Number of Abandoned Calls	Average Talk Time (minutes)	Average Handling Time (minutes)	Average Call Time (minutes)	Average Hold Time (minutes)
May 11-17, 2020	33	0	15	5.2	17.92	5.2	4.33
May 18-24, 2020	37	8	3	16.03	46.26	16.03	4.08
May 25-31, 2020	32	26	4	18.9	21.73	18.9	2.77
June 1-7, 2020	25	24	1	16.23	21.45	16.23	2.72
June 8-14, 2020	31	26	6	15.4	20.43	15.4	4.65
June 15-21, 2020	24	27	2	20.35	32.84	20.35	3.88
June 22-28, 2020	26	33	5	17.08	25.11	17.08	3.63
June 29-July 5, 2020	17	29	3	17.43	19.3	17.43	6.37
July 6-12, 2020	18	37	0	13	15.22	13	2.77
July 13-19, 2020	13	33	4	9.9	14.78	9.9	5.3
July 20-26, 2020	21	46	3	9.73	14.73	9.73	2.6
July 27-31, 2020	24	29	0	22.97	21.33	22.97	2.97
August 3-7, 2020	12	28	3	15.92	17.68	15.92	0.47
August 10-14, 2020	23	27	2	12.07	16.82	12.07	0.37
August 17-21, 2020	14	16	1	13.55	19.43	13.55	4.13
August 24-28, 2020	15	16	0	11.22	15.27	11.22	3.58
August 31-September 4, 2020	14	11	1	11.18	22.92	11.18	2.07
September 7-11, 2020	11	12	1	18.05	20.5	18.05	3.1
September 14-18, 2020	30	30	4	14.35	18.6	14.35	2.43
September 21-25, 2020	16	20	1	18.35	27.02	18.35	4.72
September 28-October 2, 2020	21	25	1	16.38	46.35	16.38	0.33
October 5-9, 2020	14	28	2	13.12	17.55	13.12	1.37
October 12-16, 2020	20	17	4	15.62	23.7	15.62	4.6
October 19-23, 2020	23	41	6	15.98	22.87	15.98	1.4
October 26-30, 2020	23	21	3	10.73	15.92	10.73	3.03
**Total**	537	610	75	368.74	555.73	924.47	77.67
**Average**	21.48	24.4	3	14.75	22.23	36.98	3.11
**Standard Deviation**	7.21	10.39	3.06	3.87	8.37	10.60	1.55
